# Salivary *Porphyromonas gingivalis* predicts outcome in oral squamous cell carcinomas: a cohort study

**DOI:** 10.1186/s12903-021-01580-6

**Published:** 2021-05-03

**Authors:** Qingli Chen, Zhe Shao, Ke Liu, Xiaocheng Zhou, Lin Wang, Erhui Jiang, Tingting Luo, Zhengjun Shang

**Affiliations:** 1 The State Key Laboratory Breeding Base of Basic Science of Stomatology (Hubei-MOST) & Key Laboratory of Oral Biomedicine Ministry of Education, School & Hospita1 of Stomatology, Wuhan University, 237 Luoyu Road, Wuhan, People’s Republic of China; 2 Department of Oral and Maxillofacial-Head and Neck Oncology, School and Hospital of Stomatology, Wuhan University, Wuhan, People’s Republic of China

**Keywords:** *Porphyromonas gingivalis*, *FimA*, Genotype, Oral squamous cell carcinoma, Fimbriae, Prognosis

## Abstract

**Background:**

Studies suggest *Porphyromonas gingivalis* (*Pg*) increased the incidence of oral squamous cell carcinoma (OSCC). However, *fimA* genotypes distribution of *Pg*, the origination of *Pg* in tissue, and its prognostic value are inconclusive. We aimed to investigate the frequency of *fimA* genotypes in OSCC patients, study the association between *Pg* and OSCC, and explore the prognostic value of *Pg*.

**Methods:**

The abundance of *Pg* in saliva from the OSCC group and the OSCC-free group was analysed by qPCR. The presence of *Pg* was explored in OSCC tissue and para-cancerous tissue by in situ hybridization. The frequency of *fimA* genotypes in saliva and OSCC tissue was determined by PCR, then PCR products were sequenced and compared. Clinical data were extracted, and patients followed up for a median period of 23 months. Clinicopathological variables were compared with the abundance of *Pg* using Pearson Chi-square test or Fisher’s exact test. The disease-free survival (DFS) rate was calculated by Kaplan–Meier method with log-rank tests**.**

**Results:**

Comparing the OSCC-free group, 95 patients with OSCC showed a high abundance of *Pg* in saliva (*P* = 0.033), and OSCC tissue showed strong in situ expression of *Pg* compared with paired normal tissue. Patients with OSCC showed a dominant distribution of *Pg* with genotype I + Ib (21.1%), II (31.6%), and IV (21.1%). *FimA* genotypes detected in saliva were in accordance with those in OSCC tissue, there was, moreover, a significant similarity in amplified *Pg* fragments. Of the 94 responsive OSCC patients, the recurrence rate was 26.6% (25/94). Overabundance of *Pg* in saliva showed advanced pathologic staging (*P* = 0.008), longer disease-free time (*P* = 0.029) and lower recurrence rate (*P* = 0.033). The overabundance of *Pg* in saliva was associated with improved disease-free survival (*P* = 0.049).

**Conclusions:**

This study indicated that *Pg* might involve in the pathogenesis of OSCC, *Pg* carrying *fimA* I, Ib, II, and IV were prevalent genotypes in patients with OSCC, the provenance of *Pg* in OSCC tissue might be from the salivary microbial reservoir, and the abundance of *Pg* in saliva might consider as a favorable potential prognostic indicator in OSCC.

## Background

There were approximately 354,864 new cases of lip and oral cavity cancer and 177,384 related deaths worldwide in 2018 [1]. Oral squamous cell carcinoma (OSCC) is the most common malignant disease in the head and neck besides non-melanoma skin cancer. Traditionally, risk factors associated with OSCC included tobacco/alcohol consumption, betel quid chewing, virus infection, dietary factor, vitamin/mineral deficiencies, occupational exposures, and heritable conditions. However, accumulating epidemiologic, clinicopathologic, and molecular studies have proven that oral microbial species played an important role in the carcinogenesis of OSCC [2].

*Porphyromonas gingivalis* (*Pg*), an anaerobic Gram-negative bacterium, was associated with a high risk of OSCC in extensive studies [3–9]. However, the effect of *Pg* infection on the prognosis of OSCC is unclear. Recently, *Pg* infection in OSCC tissue showed a trend (HR 0.34; *P* = 0.055 in multivariate regression analyses) towards improved overall survival [10]. Given that salivary microbiota is stable and saliva is easy and non-invasive to collect, saliva is an acceptable biofluid for the evaluation of the oral microbiome [11, 12]. We hypothesized that the abundance of *Pg* in saliva was associated with a favorable prognosis in patients with OSCC.

Fimbriae are crucial to initial attachment, organization of biofilms, and adhesion that mediate invasion and colonization of cells, although *Pg* has an arsenal of virulence factors, such as gingipain, lipopolysaccharide, and capsule [13]. The major fimbriae fimA and the minor one mfa1 were two distinct fimbriae expressed in *Pg*. Six genotypes (types I-V, Ib) of *fimA* have been identified based on sequence variations. Previous studies have reported a high prevalence of *fimA* genotype II in periodontitis patients, and *fimA* genotype Ib, II and, IV are more aggressive [14–17]. However, no study was found evaluating the frequency of *fimA* genotypes in patients with OSCC.

In this study, we explored the association between *Pg* and OSCC. Meanwhile, we investigated the prevalence of *fimA* genotypes in patients with OSCC. To verify the origination of *Pg* in the OSCC tissue, we compared the frequency of *fimA* genotypes in saliva and those in OSCC tissues. Furthermore, we compared the amplified *Pg* fragments from OSCC tissue and those from saliva. The potential prognostic value of *Pg* in OSCC was also assessed.

## Methods

Approval from the institutional review board was obtained at the Hospital of Stomatology Wuhan University before starting the study (2016–60). Informed consent was obtained from each patient.

The inclusion criterion was patients with primary OSCC managed by curative-intent, and a total number of 111 consecutive patients were included from October 2018 and April 2019. The exclusion criteria were patients: (1) received oral prophylaxis in the latest three months (one patient), (2) underwent radiotherapy and/or chemotherapy before surgery (three patients), (3) edentulous (two patients), (4) refused to receive surgery, (5) disagreed to participate in this study (four patients disagreed to the collection of saliva and six patients did not fast overnight before saliva sample collection).

A total number of 95 patients with OSCC (65 male and 30 female subjects, aged 21–82 years, mean age 55.8) treated in the hospital were included in this study. Pathological staging was stratified in accordance with the eighth edition of the American Joint Committee on Cancer [18]. Except for one patient with bone metastatic OSCC, all patients were M0 category. The control group comprised 39 OSCC-free subjects (21 males and 18 females, aged 33–76 years, mean age 52.6) diagnosed with salivary gland disease, lymphadenopathy, lymphoma, buccal or tongue chronic infection, epulis, ranula, lipoma, lymphoepithelial cyst, and sebaceous gland carcinoma.

Clinical records were retrieved. Assessed clinicopathological variables included age, gender, systematic disease, location of the tumor, size, pathological report, smoking, alcohol consumption, and treatment. Systemic disease included hypertension, diabetes, coronary artery disease, and chronic hepatitis B. Sixty-four patients underwent surgery, twenty-two patients underwent surgery + radiotherapy (IMRT: 76 Grays to 63 Grays) and eight patients underwent surgery + chemo-radiotherapy (Docetaxel, Cisplatin, 5-Fluorouracil). Pathological diagnosis was established by one pathologist and confirmed by another experienced pathologist from the department of pathological at the Hospital of Stomatology Wuhan University.

Patients were followed up from discharge by telephone or clinical assessment. Pathologic confirmation of recurrence was obtained in patients with clinical signs or symptoms. Disease-free survival (DFS) is defined as the time (in months) from the date of discharge to March 2021 or until the date recurrence was diagnosed.

A total of 134 saliva samples were collected between 6 a.m. and 8 a.m. following an overnight fast and refrainment of tooth brushing. Subjects were asked to swish vigorously with 40 mL sterilized double distilled water (bacteria negative in PCR assay) for 1 min, and then to expectorate into another specimen tube [19]. The saliva samples were centrifuged at 14,000 rpm for 15 min, and then the cell pellet was suspended in 1 mL of sterile TE buffer. Saliva samples stored at -80 ºC until testing.

Bacterial DNA was extracted from saliva samples using a commercial DNA extraction kit (DP302, Tiangen, China) according to the manufacturer’s protocol, except adding an enzymatic lysis step with lysozyme (20 mg/ml, 37 ºC, 60 min). The resultant DNA was stored at − 20 ºC until in PCR.

A total of 15 out of 95 fresh-frozen OSCC tissue samples were obtained. DNA was extracted using the Total DNA/RNA/Protein Kit (R6734, Omega Bio-tek, USA) according to the procedure recommended by the manufacturer. Quantification of *Pg* in saliva samples and detection of *fimA* genotypes were measured by Real-time quantitative PCR. Amplifications were performed in duplicate on Bio-Rad CFX96 thermal cycler (Bio-Rad Laboratories, USA). The primers, synthesized by Sangon Biotech (Shanghai, China), were listed in Table 1.

**Table 1:** Specific oligonucleotides used in this study

Primer	Sequence (5′-3′)	Annealing temperature (°C)	References
Universal primers	F: TCCTACGGGAGGCAGCAGT	60	[20]
	R: GGACTACCAGGGTATCTAATCCTGTT		
*Pg*	F: ACCTTACCCGGGATTGAAATG	58	[20]
	R: CAACCATGCAGCACCTACATAGAA		
*fimA* I	F: CTGTGTGTTTATGGCAAACTTC	58	[21]
	R: AACCCCGCTCCCTGTATTCCGA		
*fimA* Ib	F: CAGCAGAGCCAAAAACAATCG	58	[14]
	R: TGTCAGATAATTAGCGTCTGC		
*fimA* II	F: GCATGATGGTACTCCTTTGA	58	[22]
	R: CTGACCAACGAGAACCCACT		
*fimA* III	F: ATTACACCTACACAGGTGAGGC	58	[21]
	R: AACCCCGCTCCCTGTATTCCGA		
*fimA* IV	F: CTATTCAGGTGCTATTACCCAA	58	[21]
	R: AACCCCGCTCCCTGTATTCCGA		
*fimA* V	F: AACAACAGTCTCCTTGACAGTG	58	[14]
	R: TATTGGGGGTCGAACGTTACTGTC		
*Pg* probe used in ISH	CAATACTCGTATCGCCCGTTATTC-Digoxin		[3]

*Pg*: *Porphyromonas gingivalis*. ISH: In situ hybridization

The reaction mixture of 20 μL was composed of 50 ng saliva DNA template or 2 μL tissue DNA template, 0.4 μM of the specific primers, ChamQTM SYBR® qPCR Master with a final concentration of 1X (Q311, Vazyme, China), and an appropriate dose of sterilized DNase-RNase-free water. The conditions for Real-time quantitative PCR were as follows: 94 ºC for 5 min, then 28 cycles for *Pg* or 40 cycles for *fimA* genotypes of 30 s at 94 ºC, 45 s at 58 ºC or 60 ºC, and 1 min at 72 ºC; with a final extension of 10 min at 72 ºC. Melting curves were generated from 60 ºC to 95 ºC and read every 0.5 ºC for 5 s. An average Ct value was obtained. The ΔCt for *Pg* was determined by subtracting the Ct value of *Pg* from that of universal primer. The relative abundance of *Pg* was calculated by the 2^−ΔΔCt^ method.

Amplified PCR products of *fimA* genotype from Real-time quantitative PCR were checked on 2% agarose gel (ST004L, Beyotime, China). This was done using 1X Tris Acetate-EDTA buffer (TAE) from 50X TAE (ST716, Beyotime, China). Gels were stained with 4S GelRed (A616697, Sangon Biotech, China). Image results were captured with the digital imaging system (NuGenius, SYNGENE, UK). One pair of amplified *Pg* fragments from OSCC tissue and saliva were confirmed following nucleotide sequencing by Sangon Biotech (Shanghai, China) and the correlation of two sequences by aligning two sequences with BLAST (http://www.ncbi.nlm.nih.gov/BLAST) [23].

Among 15 OSCC tissue patients, remaining OSCC tissue and normal tissue adjacent to OSCC from one patient were fixed in 4% paraformaldehyde, paraffin-embedded, and cut into 4 μm sections, which was stained with haematoxylin and eosin, gram and subjected to in situ hybridization (ISH) using Enhanced Sensitive ISH Detection kit I (POD) (MK1030, Boster, China) according to the manufacturer's instructions. The probe is listed in Table 1. Omission of the probe was obtained as the negative controls.

### Statistical analysis

Shapiro–Wilk test was used to assess whether or not data were normally distributed. Normally distributed data were analysed by Student’s t test and presented as Mean ± Standard Deviation. The data without normal distribution presented as the median and inter-quartile range (M, Q) and analysed by the Mann–Whitney U test. Categorical variables were analysed by Pearson Chi-square test or Fisher’s exact test. The cutoff point to convert the number of *Pg* 16S rRNA gene copies into categorical data (low, < 4 and high, ≥ 4) was performed using X-tile software [24]. The Kaplan–Meier log-rank test was performed to compare the DFS. All two-tailed *P* values < 0.05 were considered significant. All analyses were carried out using IBM SPSS Statistics software (IBM SPSS Statistics V.25.0, USA).

## Results

**Fig. 1:**
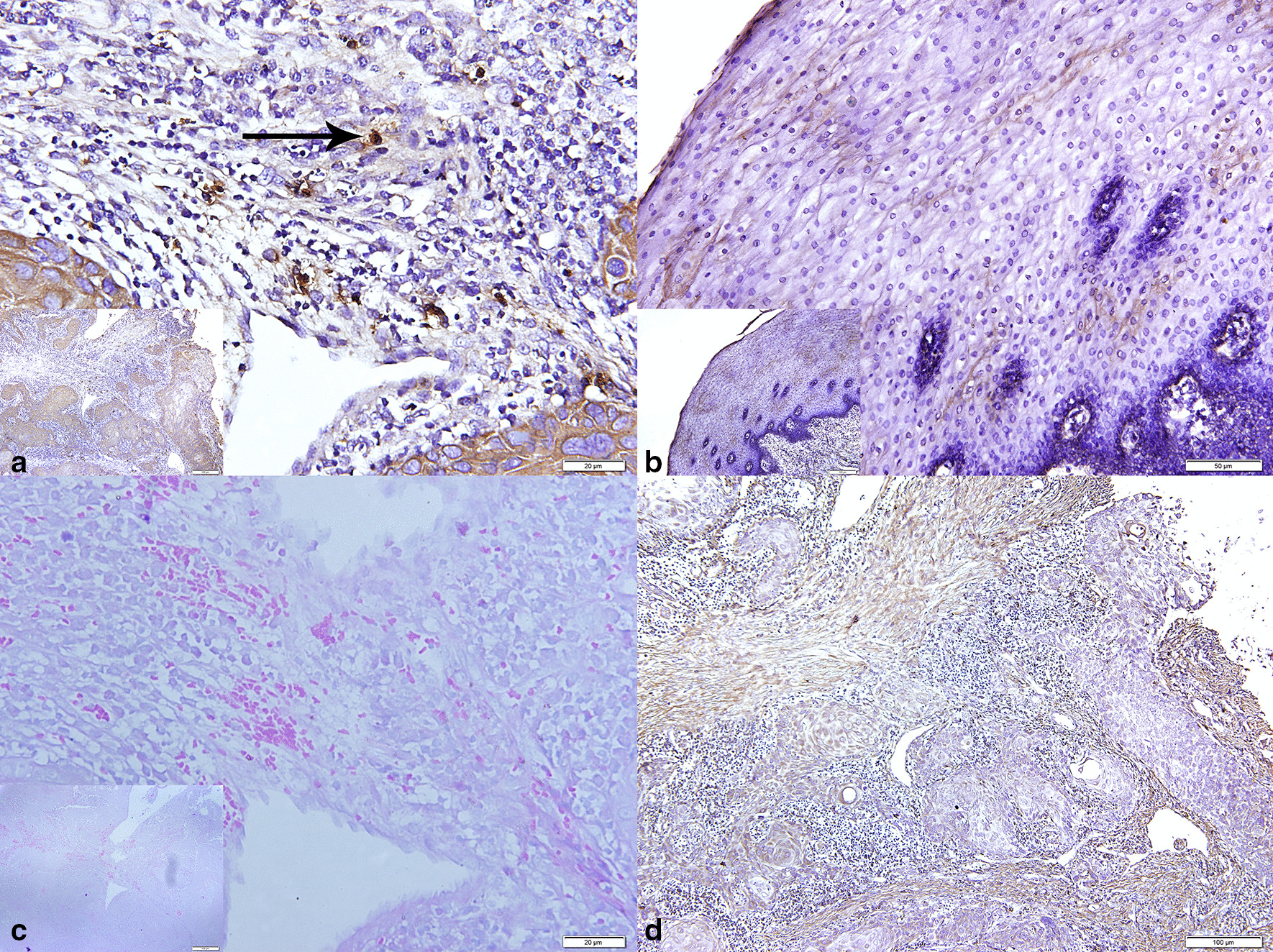
Expressions of *Porphyromonas gingivalis* in oral squamous cell carcinoma (OSCC) (**a**), normal tissue adjacent to OSCC (**b**), and probe-free as negative control (**d**) by in situ hybridization; Gram staining (**c**)

As showed in Table 2, compared with controls matched for gender and age (*P* > 0.05, respectively), OSCC patients showed an overabundance of *Pg* in saliva (*P* < 0.05). To exclude contamination of samples, *Pg* was also detected in tissues by ISH from one patient. Compared with normal tissue which is adjacent to OSCC, OSCC tissue showed strong in situ expression of *Pg* (Fig. 1).

**Table 2:** Overabundance of *Pg* in saliva from OSCC patient

	*Porphyromonas gingivalis*		*P*
	Low	High	
OSCC	84	11	0.033
OSCC-free	39	0

To evaluate the frequency of different *fimA* genotypes in patients with OSCC. Amplified PCR products of *fimA* genotype were checked on 2% agarose gel electrophoresis. The distribution of *fimA* genotype from the saliva of 95 OSCC patients was listed in Table 3. *FimA* genotype I and Ib were detected in 20 (21.1%) specimens, genotype II in 30 (31.6%) specimens, genotype III in 4 (4.2%) specimens, genotype IV in 20 (21.1%) specimens, genotype V in 2 (2.1%) specimens. We also found two or more genotypes of *fimA* from one sample. *FimA* genotype I, Ib, and II was detected in 1 (1.1%) participant, genotype I, Ib, and III in 2 (2.1%) participants, genotype I, Ib and IV in 1 (1.1%) participant, genotype I, Ib, and V in 1 (1.1%) participant, genotype II and IV in 3 (3.2%) participants, genotype I, Ib, II, and IV in 1 (1.1%) participant. Ten participants showed negative on 2% agarose gel electrophoresis assay. This finding supported the dominant distribution of *Pg* with genotype I, Ib, II, and IV in saliva from OSCC patients.

**Table 3:** Clinicopathological details

Parameters		Number (%)	*Porphyromonas gingivalis*		*P* value
			Low	High	
Age (years)	55.8 ± 12.7	95 (100)	55.7 ± 12.6	56.5 ± 14.6	0.860
Gender	Male	65 (68.4)	57	8	0.999
	Female	30 (31.6)	27	3	
Systemic disease	No	69 (72.6)	61	8	0.999
	Yes	26 (27.4)	23	3	
Pathologic staging	pStage I + II	69 (72.6)	65	4	0.008
	pStage III + IV	26 (27.4)	19	7	
Smoking	No	45 (47.4)	39	6	0.612
	Yes	50 (52.6)	45	5	
Alcohol consumption	No	57 (60.0)	50	7	0.999
	Yes	38 (40.0)	34	4	
Location	Buccal	21 (24.2)	21	2	0.447
	Tongue	52 (54.7)	47	5	
	Gingiva	11 (11.6)	9	2	
	Floor of mouth	5 (5.3)	4	1	
	Hard palate	4 (4.2)	3	1	
Differentiation grade	Well	17 (19.1)	16	1	0.735
	Moderate	65 (73.0)	57	8	
	Poor	7 (7.9)	6	1	
*FimA* genotypes	I + Ib	20 (21.1)	17	3	0.795*
	II	30 (31.6)	27	3	
	III	4 (4.2)	4	0	
	IV	20 (21.1)	16	4	
	V	2 (2.1)	2	0	
	I + Ib + II	1 (1.1)	1	0	
	I + Ib + III	2 (2.1)	2	0	
	I + Ib + IV	1 (1.1)	1	0	
	I + Ib + V	1 (1.1)	1	0	
	II + IV	3 (3.2)	3	0	
	I + Ib + II + IV	1 (1.1)	1	0	
	Untyped	10 (10.5)	9	1	
Treatment^†^	Surgery	64 (68.1)	60	4	–
	Surgery + RT or CRT**	30 (31.9)	23	7	
Outcome^†^	Recurrence	24 (25.5)	25	0	0.033
	Disease-free	70 (74.5)	58	11	
Disease-free time (months)	23, 10	94 (98.9)	22, 13.8	27, 8	0.029

*Chi-square test was used among *fimA* genotype I + Ib, II, III, IV, V

^†^One patient dropped out in follow-up

**RT: Radiotherapy, CRT: Chemo-radiotherapy

**Fig. 2:**
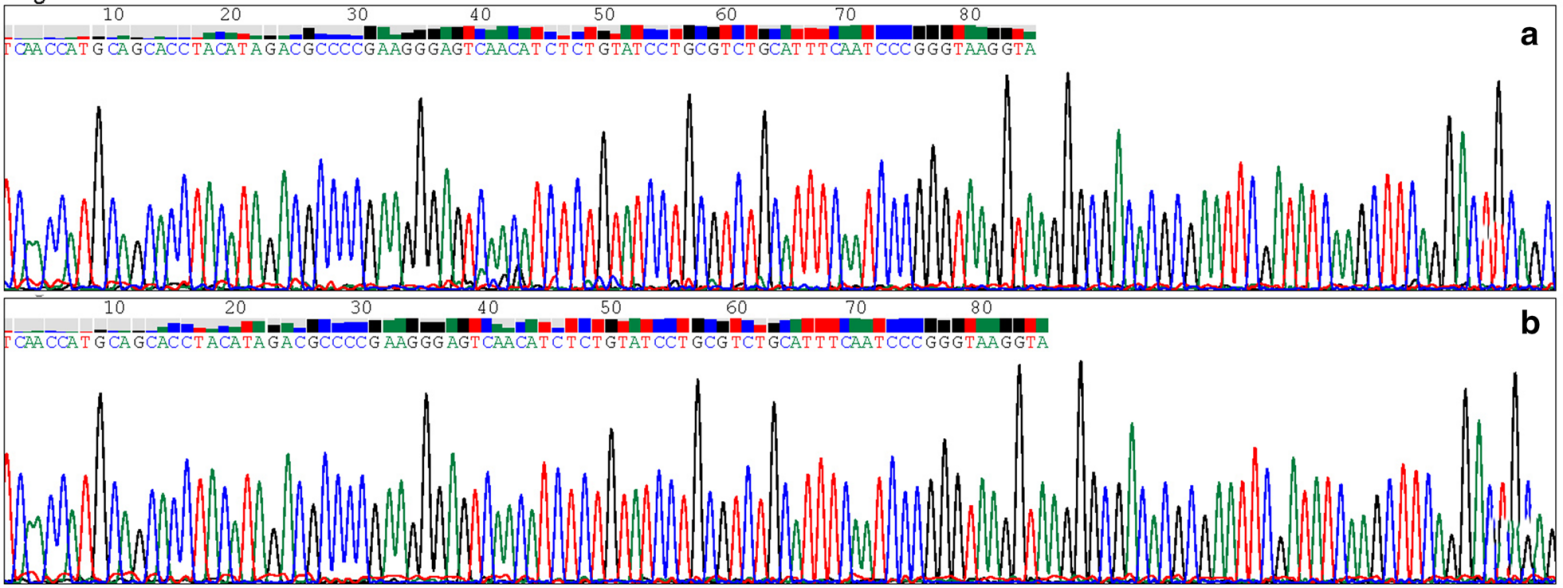
The PCR product examined *Porphyromonas gingivalis* in saliva (**a**) and oral squamous cell carcinoma tissue (**b**)

**Fig. 3:**
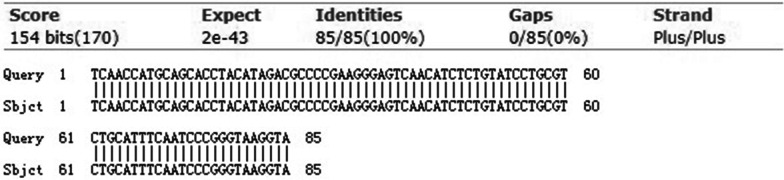
The homology analysis of *Porphyromonas gingivalis* detected in saliva and oral squamous cell carcinoma tissue

To clarify the homogeneity of *Pg* between saliva and OSCC tissue, the frequency of *fimA* genotypes was also detected in fifteen OSCC tissues. Among fifteen patients, the *fimA* genotypes detected in saliva were in accordance with those in OSCC tissue (Table 4). Besides, amplified *Pg* fragments from OSCC tissue and those from saliva were examined in one patient, we found a significant correlation in nucleotide similarity (Figs. 2, 3). Collectively, these results implied that *Pg* in OSCC tissue might originate from the salivary microbial reservoir.

**Table 4:** The frequency of *fimA* genotypes in saliva and in oral squamous cell carcinoma tissues

	I + Ib	II	III	IV	V	I + Ib + III
Saliva	3	6	1	2	2	1
Tissue	3	6	1	2	2	1

**Fig. 4:**
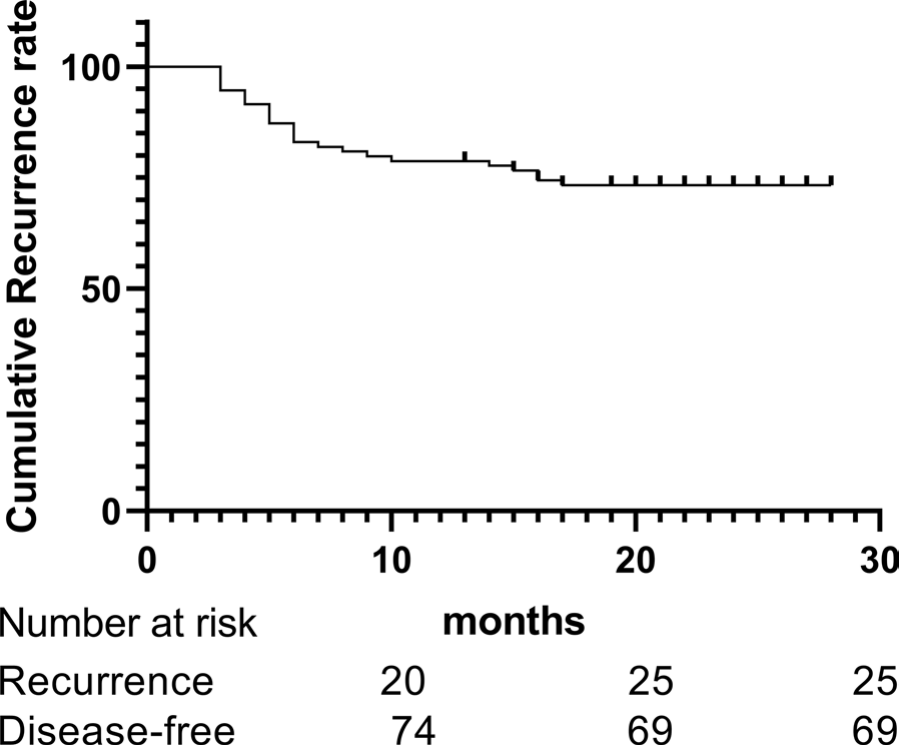
Cumulative recurrence curve of 94 patients

The deadline for follow-up was March 2021. After a median follow-up period of 23 months (range 3 to 28 months). Ninety-four patients were available for the follow-up visit, but one patient was non-responsive to any form of contact. Recurrence was diagnosed as the endpoint for 25 patients, with a 26.6% (25/94) cumulative recurrence rate (Fig. 4).

Clinicopathological information of OSCC patients is shown in Table 3. The distribution of clinicopathological outcomes was compared with the abundance of *Pg* to assess the potential prognostic variables. Patients with the overabundance of *Pg* in saliva had an advanced pathologic staging than those with a low abundance of *Pg* (*P* = 0.008). While, compared with the weak group, patients with the overabundance of *Pg* in saliva had a longer disease-free time (*Z* = -2.188, *P* = 0.029). The overabundance of *Pg* in saliva was associated with a lower recurrence rate (*P* = 0.033). However, differences were not statistically significant by age, gender, systemic disease, smoking, alcoholic consumption, location, differentiation grade, and *fimA* genotypes. Neither single *fimA* genotype was statistically significantly associated with any clinicopathological parameters.

**Fig. 5:**
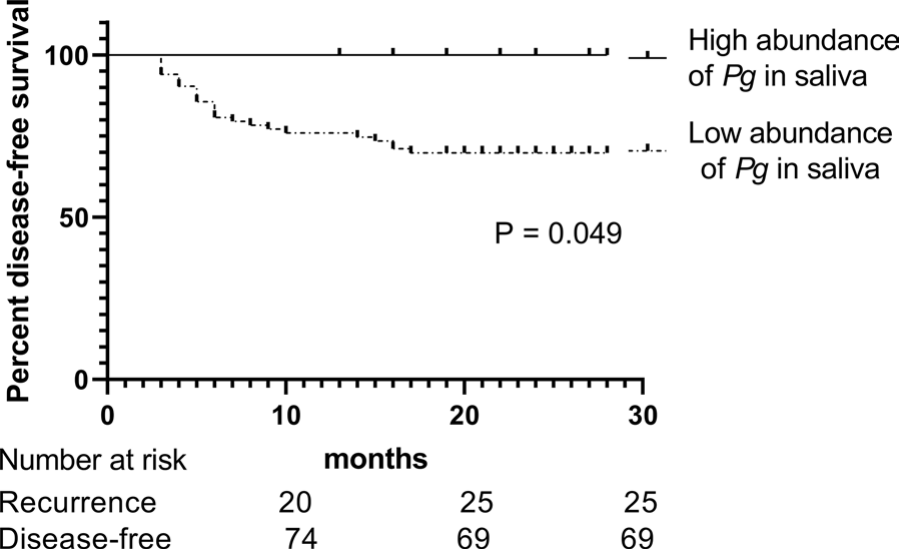
Disease-free survival according to 94 oral squamous cell carcinoma patients at the time of leaving the hospital. The Kaplan–Meier method for recurrence with the log-rank test was used for statistical comparisons

A total of 94 cases with follow-up data were included in the survival analysis. Univariate analysis showed that the overabundance of *Pg* was a favorable prognostic factor (*Chi-square* = 3.86, *P* < 0.05) (Fig. 5). Statistically, age, gender, *FimA* genotypes, systemic disease, pathologic staging, smoking, alcoholic consumption, location, differentiation grade, and treatment were not the independent prognostic indicators. Consequently, our results showed the overabundance of *Pg* was associated with favorable outcomes in patients with OSCC.

## Discussions

With recent breakthroughs in high-throughput genetic-based tools, there has been a hot issue concerning the relationship between the oral microbiome and neoplasms, especially OSCC. Recently accumulating evidence indicated the relationship between *Pg* and OSCC. Immortalized oral keratinocytes stimulated with *Pg* led to a more aggressive malignant profile phenotype and contributing to enhanced tumor features [25]. The serum immunoglobulin G antibody against *Pg* was higher in OSCC patients compared with non-OSCC patients [4]. *Pg* increased the size and the multiplicity of carcinoma to promote the development of oral cancer [5]. Previous studies have confirmed the association between *Pg* and OSCC by examining the abundance of *Pg* in the saliva of patients and unveiled that patients with medium and poor differentiation, overall clinical stage III and stage IV, lymph node metastasis, and shorter overall survival associated with *Pg* involvement [3, 10]. But in multivariate analyses, colonization of *Pg* was a favorable prognostic factor, with a strong tendency towards statistical significance (HR 0.340, 95% CI 0.112–1.025, *P* = 0.055) [10]. Our study also verified the overabundance of *Pg* from OSCC patients compared with OSCC-free subjects in saliva, and the overabundance of *Pg* in saliva was more likely to correspond with the advanced pathological stage. Those results are in resonance to previous reports [3]. To exclude contamination of samples, we examined the presence of *Pg* in OSCC tissue by ISH. Consistent with the previous report [3], there was high enrichment of *Pg* in OSCC tissue compared with normal tissue adjacent to OSCC. After a median follow-up period of 23 months, the OSCC recurred in 26.6% (25/94) of patients. As the same as our findings, it was reported early-stage patients have a 90–95% survival rate for one year or more, and advanced-stage patients have a 65–70% survival rate [26].

In this study, compared with the weak group, patients with the overabundance of *Pg* in saliva had a longer disease-free time and lower recurrence rate. Those suggested that *Pg* may affect the prognosis of OSCC. Most interestingly, we found the overabundance of *Pg* is a favorable prognostic factor for DFS. Contrary to popular belief, they found that *Pg* was associated with a higher risk of pancreatic cancer [27, 28], esophageal squamous cell carcinoma [29, 30], and oral squamous cell carcinoma [3, 10]. Meanwhile, *Pg* was associated with poor overall survival rates in esophageal squamous cell carcinoma [31]. Furthermore, patients with a high level of *Pg* had the worst prognosis in esophageal squamous cell carcinoma [30]. Besides the population and follow-up period contributed to this prognostic incongruity, the inherent mechanistic also should be taken into consideration.

One of the most vital virulence in *Pg* has been supposed to the presence of fimbriae, which plays an important role in adhesion, colonization, and invasion to tissues [32]. Most of the studies focused on the distribution of *fimA* genotypes in periodontitis. However, the frequency of *fimA* genotypes in OSCC was not clear. *FimA* genotypes I and Ib could be discriminated by Rsa I enzyme digestion. However, discrimination of genotypes I and Ib seems to be improbable [14]. Besides, there were no differences in the immunological analysis between *fimA* I and Ib fimbriae [14]. So we consider *fimA* genotypes I and Ib as a whole.

In this study, the association of *fimA* genotypes and clinicopathological parameters was not statistically significant. However, the predominantly detected *fimA* genotypes in OSCC were genotypes I, Ib, II, and IV. Several studies concluded that nucleotide genetic variation was likely associated with virulence. Some reported *fimA* genotypes Ib, II, and IV are the most virulent fimbriae in periodontitis and assist in adhesion and invasion [14, 33]. It was reported that *fimA* genotype Ib, II, and IV led to more severe infections and inflammations [34, 35]. Clinical isolation of *Pg* from chronic periodontitis patients also supported the virulence of *fimA* genotypes Ib, II, and IV [36]. Different *Pg fimA* genotype was injected subcutaneously, and Nakano et al. found that the weakest inflammatory response was induced by genotype III [35]. *FimA* genotype V was the least amount of genotypes in this study. The reason might be the low prevalence (0–29%) of this genotype [37]. Single *Pg fimA* genotype was determined by more than 70% of OSCC patients, and two or more genotypes were also detected in a subset of the subjects. Approximately 10% of the samples were multiple genotypes in this population, which was less than the results of other studies [14, 17]. Researchers attributed to the limitations of PCR in the discrimination of *fimA* genotypes and the possibility of classifying new genotypes [38].

Due to the conservative properties of DNA, the bacterial 16S ribosomal DNA allows identification of the genus and species. Analysis of *Pg* nucleotide sequences in the OSCC tissue and those in the saliva showed a homology of 100%. Moreover, the distribution of *fimA* genotypes in OSCC tissue is according to those in saliva. Those results support the origination of *Pg* in OSCC tissues might be from the salivary microbial reservoir [39].

The limitations of this study included the short follow-up period and small sample. Further research will need to be done to elucidate the mechanism of the prognostic role. Except for fimbriae of *Pg*, important virulence included: encapsulation (K1–K6), gingipains (types A, B, C) as well as lysine-specific types I and II, may also involve in the carcinogenesis of OSCC. Those remain to be uncovered in future studies.

## Conclusions

This study found the overabundance of *Pg* was associated with OSCC, and patients with advanced pathological stage, longer disease-free time, and the lower recurrence rate were related to the overabundance of *Pg*. Meanwhile, the overabundance of *Pg* was a favorable prognostic factor in patients with OSCC. Furthermore, there was a dominant distribution of *Pg* with genotype I, Ib, II, and IV from patients with OSCC, and the origination of *Pg* in the tumor might be from the salivary microbial reservoir.

## Data Availability

The raw data are confidential and cannot readily be shared. Researchers need to obtain permission from the Institutional Review Board and apply for access to the data from The Ethics Committee of Stomatological Hospital, Wuhan University.

## Abbreviations

OSCC: Oral squamous cell carcinoma
*Pg*: *Porphyromonas gingivalis*
ISH: In situ hybridization
